# A Video Interview With Robert Singer, MD, FACS: My Second Thoughts About the Coronal Browlift

**DOI:** 10.1093/asjof/ojac092

**Published:** 2022-12-21

**Authors:** Robert Singer, Lorne King Rosenfield

**Affiliations:** The University of California, San Diego, San Diego, CA, USA; Department of Plastic Surgery, University of California San Francisco, San Francisco, CA, USA

Welcome to another edition of *Second Thoughts on First Thoughts* where we ride the learning curve of one of our expert colleagues. In this session, we chat with one of the true leaders in our field, Robert Singer (Video). He talks about how he has moved beyond the classic open coronal forehead/brow lift to a palette of “kinder, gentler” strategies and still achieved similar outcomes.

The interview follows the format of the 5 Ws in investigative reporting: Who, What, Where, When, and Why? as a shout-out to the longest-running Canadian TV show of its kind, “W5.” We hope you enjoy this brief but enlightening interview.

## Supplementary Material

ojac092_Supplementary_Data

